# Cross-cutting view of current challenges in paediatric solid organ and haematopoietic stem cell transplantation in Europe: the European Reference Network TransplantChild

**DOI:** 10.1186/s13023-020-1293-0

**Published:** 2020-01-15

**Authors:** P. Jara, A. Baker, U. Baumann, A. M. Borobia, S. Branchereu, M. Candusso, A. J. Carcas, C. Chardot, J. Cobas, L. D’Antiga, C. Ferreras, E. Fitzpatrick, E. Frauca, F. Hernández-Oliveros, P. Kaliciński, C. Lindemans, M. F. Lopes, E. López-Granados, C. de Magnée, C. Mota, J. M. Muñoz, J. J. Ojeda, A. Pérez-Martínez, G. Perilongo, J. Rascon, M. Sciveres, R. Stone, V. Tarutis, J. Toporski, J. M. Torres, L. Wennberg, On behalf of ERN TransplantChild

**Affiliations:** 1 Institute for Health Research (IdiPAZ), La Paz University Hospital, Madrid, Spain; 2 Pediatric Hepatology Department, La Paz Children’s University Hospital, Madrid, Spain; 3 Pediatric Liver, GI and Nutrition Centre, King’s College Hospital, Denmark Hill, London, UK; 4 Department for Pediatric Kidney, Liver, and Metabolic Diseases, Division of Pediatric Gastroenterology and Hepatology, Hannover Medical School, Hannover, Germany; 5 Clinical Pharmacology Department, La Paz University Hospital, Madrid, Spain; 6 Pediatric Surgery Department, Hospital du Kremlin Bicêtre, Paris, France; 7 Division of Hepatology and Gastroenterology, Bambino Gesù Children’s Research Hospital IRCCS, Rome, Italy; 8 Pediatric Surgery Department, Hospital Necker enfants malades, Paris, France; 9 La Paz University Hospital, Madrid, Spain; 10 Centre for Pediatric Hepatology, Gastroenterology and Transplantation, Hospital Papa Giovanni XXIII, Bergamo, Italy; 11 Paediatric Liver Centre, King’s College London at King’s College Hospital, London, UK; 12 Pediatric Surgery Department, La Paz University Hospital, Madrid, Spain; 13 Department of Pediatric Surgery and Organ Transplantation, Children’s Memorial Health Institute, Warsaw, Poland; 14 Pediatric Blood and Marrow Transplantation Program, University Medical Center, Utrecht, Netherlands; 15 Department of Pediatric Surgery, Pediatric Hospital, Centro Hospitalar e Universitário de Coimbra, Coimbra, Portugal; 16 Department of Clinical Immunology, La Paz University Hospital, IdiPAZ, Madrid, Spain; 17 Department of Pediatric Surgery, Cliniques Universitaires St Luc (Bruxelles-Université Catholique de Louvain), Saint-Luc University Hospital, Brussels, Belgium; 18 Department of Paediatric Nephrology, Paediatric Service, Centro Materno Infantil do Norte, Centro Hospitalar do Porto, Porto, Portugal; 19 Pediatric Hemato-Oncology, La Paz University Hospital, IdiPAZ, Madrid, Spain; 20 Department of Pediatrics, University Hospital of Padua, Padua, Italy; 21 Center for Pediatric Oncology and Hematology, Children’s Hospital, Affiliate of Vilnius University Hospital Santaros Klinikos, Vilnius, Lithuania; 22 Pediatric Hepatology and Liver Transplantation, ISMETT UPMC Palermo, Palermo, Italy; 23 Unidade de Nefrologia e Transplantação Renal Pediátrica. Hospital de Santa Maria, Centro Hospitalar de Lisboa Norte, Lisbon, Portugal; 24 Centre of Cardiac Surgery, Vilnius University Hospital Santariskiu Klinikos, Vilnius, Lithuania; 25 Department of Pediatrics, Skåne University Hospital, Lund, Sweden; 26 Department of Transplantation Surgery, Karolinska University Hospital, Huddinge, Sweden

**Keywords:** Paediatric, Transplantation, Solid organ transplantation, Surgical procedures, Haematopoietic stem cell transplantation, Post-transplant management, Quality of life

## Abstract

The low prevalence of European paediatric transplanted patients and scarcity of resources and expertise led to the need for a multidisciplinary network able to improve the quality of life of paediatric patients and families requiring a solid organ or haematopoietic stem cell transplantation. The European Reference Network (ERN) TransplantChild is one of the 24 ERNs established in a European legal framework to improve the care of patients with rare diseases. ERN TransplantChild is the only ERN focused on both solid organ and haematopoietic stem cell paediatric transplantation, based on the understanding of paediatric transplantation as a complex and highly specialised process where specific complications appear regardless the organ involved, thus linking the skills and knowledge of different organ disciplines. Gathering European centres of expertise in paediatric transplantation will give access to a correct and timely diagnosis, share expertise and knowledge and collect a critical mass of patients and data that increases the speed and value of clinical research outcomes. Therefore, the ERN TransplantChild aims for a paediatric Pan-European, Pan-transplant approach.

## Background

Paediatric transplantation (PT) is the only curative therapeutic procedure for most end-stage diseases affecting different organs and body systems, achieving transformative impact on the health and quality of life (QoL) of children. Between 2012 and 2016, 7.741 solid organ transplants (SOT) were performed on children in 23 European Union (EU) countries [1], increasing to 1.294 in children of up to 15 years old in 2016 in 28 EU states [2]. In 2012 a total of 4.041 paediatric patients received haematopoietic stem cell transplant (HSCT) in 28 EU and 10 affiliated states [3]. This means that more than 5.000 children receive one type or the other transplant in Europe each year [4]. Both, SOT and HSCT offers the chance of a cure, but at the same time raise the risk of treatment-related mortality and long-term side effects.

During the last 40 years the development and improvement in surgical techniques, anaesthetic procedures and post-transplant care, especially immunosuppressive strategies while increasing the number of new indications for which transplantation is required, have increased the rate of graft survival, reduced complications and improved recipient outcomes [5]. Due to those improvements, the primary burden of disease in transplanted children has shifted to a chronic condition related to side effects from immunosuppression to avoid rejection, and the requirement for proper monitoring and care of post-transplant complications such as infections, malignancy and chronic graft dysfunction. This chronic condition and its management is even more important in children as they aspire to a life expectancy of up to 8 decades, which is the current normal in Europe. However, despite improvements in short-term graft survival, current immunosuppressive regimens are still associated with somewhat limited long-term graft survival, estimated between 5 and over 20 years depending on the transplanted organ [6].

### Challenges in paediatric transplantation

Paediatric surgical procedures allow transplantation of virtually all organs but there remains a need for the development of new interdisciplinary knowledge. The clinical consequences of transplantation can be understood as those that are directly related to the transplanted organ (acute or chronic allograft rejection, native disease within the allograft, surgical complications), those that arise primarily from post-transplant therapies (infection, malignancy, pharmacological toxicity, growth retardation), those linked to the underlying disease (recurrence of original disease in some allografts), and those that are multifactorial. Transplanted children shift their primary disease to a chronic condition of immunosuppression to avoid rejection, requiring a proper monitoring and handling of post-transplant complications, which leads to a series of challenges specific for children (Table 1) that demands the multidisciplinary coordinated efforts of organ-focused specialists, especially when more than one organ is transplanted.

**Table 1:** Main challenges identified in Paediatric Transplantation

Main challenges	Description	Challenges classification and terms
Post-transplant morbidity and mortality	Mortality and morbidity rates are still high in children	Clinical - short-term
Longer life expectancy in children	Children poses a greater risk of prolonged and severe side effects related to long-term immunosuppression, disabilities and secondary cancer [7, 8]	Clinical – medium/long-term
Physiological Immaturity	Many organs and body systems, specially the immune, metabolic and endocrinology systems, impact on the growing and developmental process	Clinical – medium/long-term
Risk factors during adolescence	Disruptions in the continuity of medical provision during such a delicate developmental period.	Social – medium/long-term
Transition to adulthood	Additional transitional programmes are needed in order to ensure active collaboration between paediatric and adult transplant programs.	Clinical – medium/long-term
Psychosocial progress and social integration	Severe psychological and socio-economic issues could be identified during the transplant process.	Social – medium/long-term
Health-related quality of life (HRQoL) acceptable but lower than their healthy peers	HRQoL of patients and parents that taking care of transplanted children can be seriously impaired, mainly in the ability to perform tasks of daily living, to fulfil social roles, and the psychological well-being of the patients.	Social – medium/long-term

Diagnostic and therapeutic advances achieved in adult transplant are therefore not necessarily one on one applicable to PT and require their strategy of development for children and young people, focusing on developmental needs, transition to adulthood and the ultimate needs of transplanted patients when they become adults perhaps decades into the future. Specialised clinical and laboratory resources that support the transplantation procedure during management and assessment of immunosuppression, immune reconstitution, rejection, tolerance, risk of infection and second malignancies, which are common to the transplantation of various organs are needed to accelerate research and new therapies [9, 10]. Also, development of new areas of non-medical supportive care is needed for children and their families, due to severe socio-economic and psychological issues that too frequently extend into adulthood, compromising the child’s chances of success in life. Paediatric disease affects the whole family as an impairment of a child’s function are a source of emotional distress for parents. Parents of transplanted children have been found to show posttraumatic stress disorder, feeling particularly distressed during the post-transplantation phase [11], but with effects persisting, if not treated. These problems may affect the child’s daily-life function with a broad spectrum of somatic, psychological, and social problems.

Conversely, PT represents an opportunity for achieving significant advances in the field of transplantation for several reasons:

ABO incompatible transplants have been performed with great success in children younger than 1 year without the need of desensitisation which is actually almost impossible in adults.

Operational tolerance is currently being pursued by different strategies in transplantation that could be favoured by the plasticity of children immune system. Recent data suggest haematopoietic chimerism as an effective strategy to induce functional tolerance, therefore, there is necessary the closer collaboration of HSCT and SOT teams, as is already perceived in most of reference centres.

The long-term follow-up inherent to PT would allow to perform clinical trials, psychosocial and quality of life studies that are not possible in adults.

Thus, the rarity of PT demands to centralise more and specific development of expertise in reference centres connecting multidisciplinary medical skills, transfer of knowledge and innovative medicine. Research and dissemination of knowledge will provide adequate care including both medical and social care working in close collaboration with a focus on recognising the organisational and psychological needs of young people transitioning from paediatric to adult services.

### The cross-cutting approach in PT

Clue challenging areas to address for further improvement in PT, are: 1) the surgical techniques and care procedures; 2) post-transplantation care with special focus in harmonisation and standardisation; 3) the mechanisms associated with graft tolerance vs rejection; 4) the prevention of complications related to long-term immunosuppression; 5) the further understanding of the specificities associated with this population group and the disease type; 5) the social and economic impact of these surgeries on patients, their families and health systems; 6) the patients and families’ empowerment. These areas are common in different types of transplants; therefore, a holistic procedure with a cross-cutting approach will improve the expectancy and long-term quality of life in children and their families, diminishing the burden of care of transplantation as a chronic long-life condition in the patient and their caregivers.

Ensuring permanent efforts for the routinely implementation and standardization of the most recent improvements in the transplantation process (at pre-transplant, transplant, and post-transplant stage) will benefit all the transplanted children population. These efforts are directly focussed on prevention, improvement of the surgical and preparative procedures, prevention of complications and secondary diseases related to transplantation, and psychosocial care and education.

**Fig. 1:**
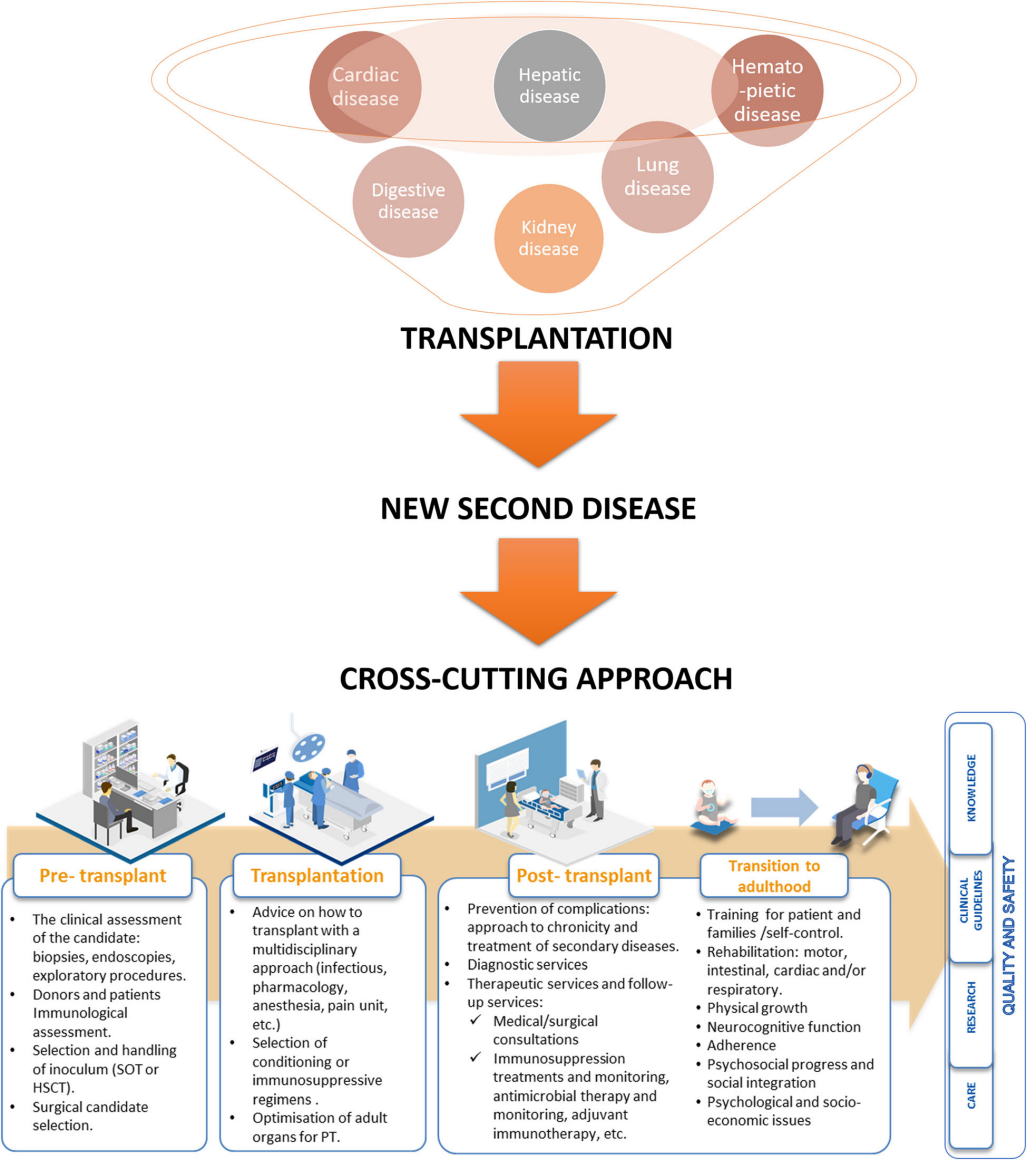
Cross-cutting approach in the transplantation process. Legend: Transplantation as a need funnel where diseases merge to be transformed in a second new disease

The cross-cutting approach will allow the identification of common topics to all types of transplants such as clinical, personal, and socio-economic issues (Fig. 1). Also, will allow the improvement of the transplant quality of life of children and their families by preventive practices to anticipate and minimise patients risks, ensuring treatment standardisation, harmonisation of clinical best practices, with more care humanisation. An improvement to obtain relevant data that will support innovation due to the higher amount of transplant cases where common complications are the focus. This view is specially needed in PT as compared to adult transplantation in which the number of patients per transplanted organ is higher.

### ERN TransplantChild

In March 2017, 24 European Reference Networks (ERN) were launched by the European Union (EU) [12, 13, 14]. ERNs are virtual networks involving healthcare providers across Europe created to tackle rare diseases and complex conditions requiring highly specialised healthcare. ERNs gather knowledge, expertise and contribute to the healthcare improvement of their patient group overcoming many of the challenges related to rare and diseases derived from highly specialised procedures. ERNs support the equity in access to prevention, diagnosis and care for patients within the EU. Patients are few and often geographically isolated, data are also scarce, and research is fragmented, resulting in limited effective treatment and therapies risking poor outcome for patients, especially in small countries where it may not be efficient or effective or even possible to develop high-quality services for all rare conditions. The pooling of resources through ERNs will overcome these barriers improving the medical care of these patients and ensuring equity in access to care across the EU.

ERN on paediatric Transplantation (ERN TransplantChild) is one of the 24 ERNs established in a European legal framework. ERN TransplantChild is the only ERN focused on a complex and highly specialised process, the PT of both SOT and HSCT as a low-prevalence condition that requires highly specialised expertise and resources (ERN TransplantChild dose not include primary diseases or conditions that lead to or indicate the need of PT, and allocation of organs for the purpose of organ transplants as established in the directive 2011/24EU). The network addresses the entire transplant process based on a crosscutting approach, dealing with all aspects of the new chronic condition represented by the management of the newly transplanted organ and its associated risks and complications. It provides a unique opportunity for healthcare professionals in PT to work across the border in Europe and between specialities to increase the exchange of knowledge, expertise and resources.

Other initiatives such as Starzl Network for Excellence in Paediatric Transplantation unite top paediatric centres in the USA and Canada but aiming to improve the outcomes of a liver transplant. Most of the initiatives are national, single organ focused including organ matching and allocation and they are not specifically paediatric. ERN TransplantChild is the first initiative worldwide where different centres of expertise from different countries join in order to tackle the hurdles of paediatric transplantation with a transversal view of both haematopoietic and solid organ transplantation.

### ERN TransplantChild: aims, strategic lines, organisation and benefits

**Fig. 2:**
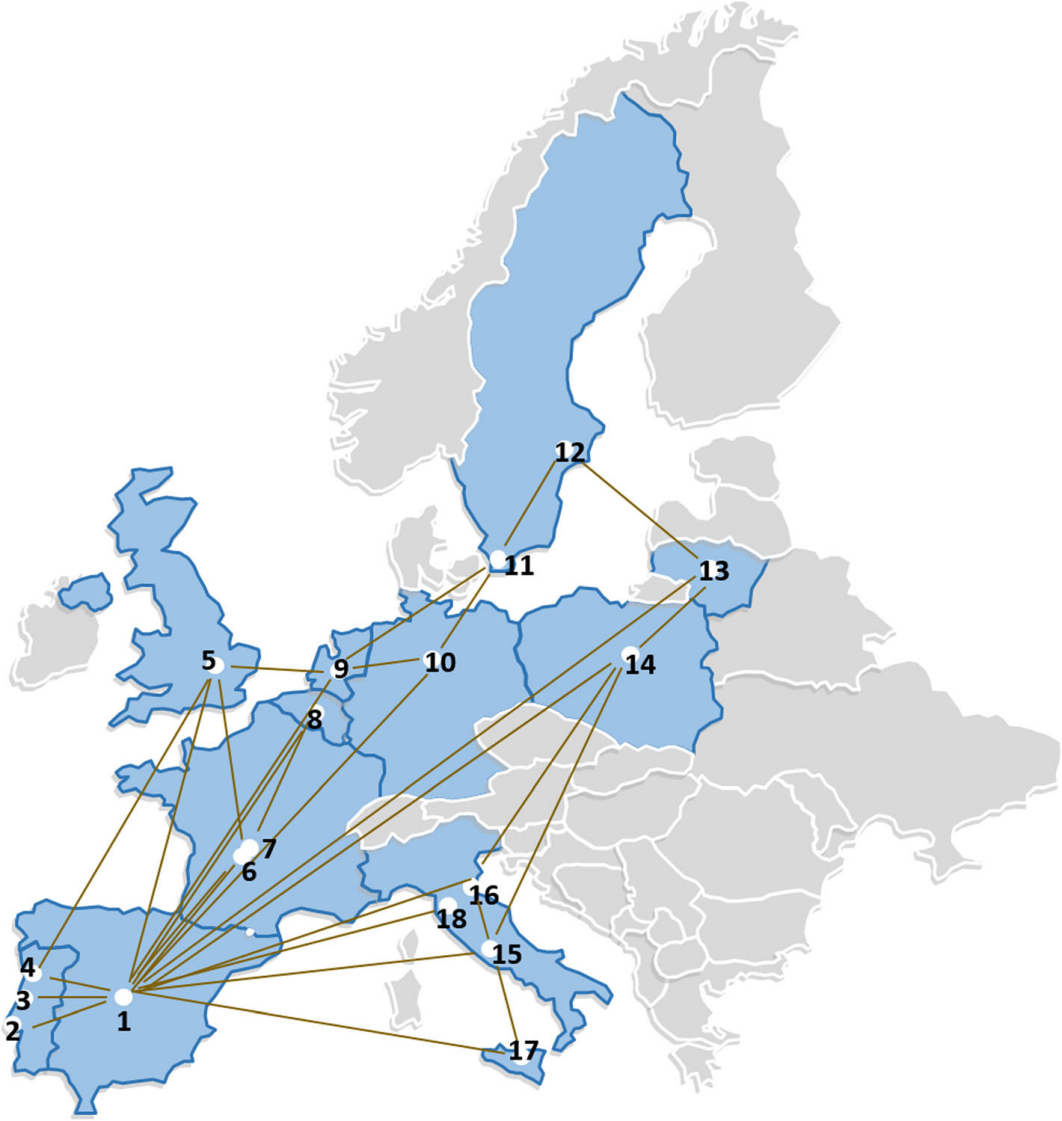
2019 map of Healthcare providers ERN TransplantChild members. Legend: Members countries and affiliation: **Spain**: 1. La Paz University Hospital. Coordinator centre; **Portugal**: 2. Hospital Santa María. Centro Hospitalar Lisboa Norte: 3. Centro Hospitalar e Universitário de Coimbra; 4. Centro Hospitalar do Porto; **United Kingdom**: 5. King’s College KCH Trust; **France:** 6. Bicêtre Hospital – Paris; 7. Necker Hospital- Enfants Malades; **Belgium**: 8. Cliniques Universitaires St Luc; Netherlands: 9. Prinses Máxima Centrum Utrecht; **Germany**: 10. Hannover Medical School; **Sweden:** 11. Children’s Hospital, Skåne University Hospital; 12. Karolinska University Hospital; **Lithuania:** 13. Vilnius University Hospital Santariskiu Klinikos; **Poland:** 14. Children’s Memorial Health of Warsaw**; Italy:** Ospedale Pediátrico Bambino Gesù; 16. Azienda Ospedaliera di Padova; 17. ISMETT, University of Pittsburgh Medical Center; 18. Ospedale Papa Giovanni XXIII

Currently, ERN TransplantChild is integrated by 18 healthcare providers from 11 Member States of the EU (Fig. 2) and will have the incorporation of new member and affiliated centres to the network that applied to the calls during 2019. The network aims the empowerment and the improvement of life expectancy and quality of life for EU paediatric patients and patients’ families requiring SOT or HSCT, by: 1) ensuring their access through the network to the best possible care practices and support procedures related to a transversal and multidisciplinary approach to children’s transplant; 2) developing and gathering efforts within the network for integrative, innovative and better procedures, information, training, knowledge and expertise; 3) integrating stakeholders in the transplantation process and making available the knowledge and information. Six strategic areas have been identified in order to comply with the mission for the network and achieve its vision (Table 2).

**Table 2:** Strategic areas, objectives working groups in ERN TransplantChild

Strategic areas	Related objective	Working group
Improve patient healthcare	Ensuring equity, transparency and coordination at local, regional and European level in order to allow the patient and family access to the best and continuous care throughout the network by using mechanisms of coordination and communication.	Healthcare
Harmonise clinical best practices	Discussing new evidence-based and standardising practices for the whole transplant process by the development of clinical practice guidelines.	Clinical practice guidelines
Harmonise research and innovation	Identifying, aligning and prioritising research area gaps focused on facilitating continuous improvement transplanted patient care and Health outcomes.	Research
Spread knowledge	Exchanging and disseminating knowledge and best practices within and outside the network and closely collaborating with other centres and Networks at both national and international level.	Networking and Knowledge management
Education and training	Identifying and fulfilling educational, training, and professional development gaps in PT, promoting the use of standardized continuous education training programmes.	Education and training
Network organisation, quality and safety	Defining the mechanisms needed for planning monitoring and reviewing the strategic approach and operating rules in order to achieve the established objectives, with the support of high quality and safe care for patients and their families.	Quality and Safety

ERN TransplantChild has assigned each of the strategic areas to a different working group (WG). Each WG is composed by members from all ERN centres, with a Technical Director from a different centre of international standing and a Coordinator from the coordinator centre (Table 3). It is expected that all WGs interact with each other. This systematic is aimed at defining the required concrete and concise activities, obtaining reliable information about their progress and facilitating their monitoring and control by the Board of the Network (BoN). The WG coordinators are responsible for organising and distributing the activities within each WG, being developed during the WG meetings as mainly activity; they are also responsible for establishing synergies with the other WGs and ensuring information and participation among the groups. The participation of patients in the activities of the working groups and the network will be ensured by the patient subcommittee. Representatives of the patient subcommittee will participate in the working groups as a full member. All the activities developed are presented in periodic meetings of the network, with the relevant decisions taken by the BoN.

**Table 3:** Technical Director and Coordinators of each WG within ERN TransplantChild

Working Group	Technical Directors/ Centre (Country)	WG Coordinators (From La Paz University Hospital Coordinator centre)
Healthcare	Alastair Baker / King’s College London (United Kingdom)	Esteban Frauca
Clinical practice guidelines	Lorenzo D’Antiga / Ospedale Papa Giovani XXIII (Italy)	Antonio J. Carcas and Alberto M. Borobia
Quality and Safety	Lars Wennberg / Karolinska Institute (Sweden)	José Jonay Ojeda
Education and Training	María Francelina Lopes / Centro Hospitalar e Universitário de Coimbra (Portugal)	Antonio Pérez Martínez
Knowledge Management and Networking	Jelena Rascon / Vilnius University Children’s Hospital (Lithuania)	Paloma Jara and Francisco Hernández
Research	Ulrich Baumann / Hannover Medical School (Germany)	Eduardo López Granados

### Added value of the network: benefits

ERN TransplantChild will be become a reference facility for all transplant centres and their patients and families in Europe, by knowledge-sharing and expertise scattered across Europe with multidisciplinary teams following the pan-transplant inclusive approach. This will result in the following benefits:

Better chances for transplanted children to receive accurate advice on the best treatment and support for their specific condition.

- ✔ Access for healthcare providers to a much larger pool of expertise
- ✔ Facilitating the patients’ access to care, improving their healthcare outcomes and the sustainability of this complex procedure for national healthcare systems, impacting positively on the sustainability of the national healthcare systems in the EU.
- ✔ Involvement of patients’ associations in education and training, communication activities and development of good practice clinical guidelines. The voices of children living with a transplant must be heard and included in the strategic and operational delivery of the network.
- ✔ Collaboration and knowledge exchange with other organ-focused or paediatric ERNs will help to create synergies for treatments, improve clinical research outcomes and increase number and quality of scientific publications. Patients whose conditions require a transplant and already transplanted patients will benefit from the sharing of professional knowledge across Europe. The individual cases particularly challenging will benefit from the interaction within the ERN community through the Clinical Patient Management System (CPMS) platform via expert consultations.
- ✔ Prolonging the life of graft survival, proper management of immunosuppression regimens or finding tolerance through new cell therapies should be at the forefront of clinical research and collaborations to increase paediatric transplanted patient survival and QoL. In clinical research, interaction with the European Medicines Agency (EMA) will open opportunities to enable ERNs to perform multicentre clinical research allowing large clinical studies or clinical trials to be undertaken rapidly to a high standard. Collaboration with other European initiatives and other relevant research networks in Europe will increase research potential. To achieve this objective, the development of a registry of paediatric transplant patients across Europe seems mandatory.

### Improving health care by IT tools: clinical patient management system

ERNs were created to ensure equity of access to care for patients with rare diseases or complex condition. To enable and facilitate this, the European Commission together with the ERNs set up the eHealth Digital Service Infrastructure for the ERNs. The Clinical Patient Management System (CMPS), has been created. CPMS is an innovative and inter-operational web-based platform through which a network coordinator can convene ‘virtual’ advisory boards of medical specialists using telemedicine tools to review a patient’s condition for diagnosis or treatment. That allows healthcare providers in the ERNs to provide and receive advice on challenging cases by using a unique Europe-wide consultation technology, responding to the most advanced EU standards in terms of security and data protection. This IT tool will allow TransplantChild to spread knowledge and expertise without the need for patients to travel. Also, this virtual system will address the problem of equity of access to specialist services and overcome financial obstacles to better care, reducing costs and optimizing outcomes and QoL throughout the EU.

**Fig. 3:**
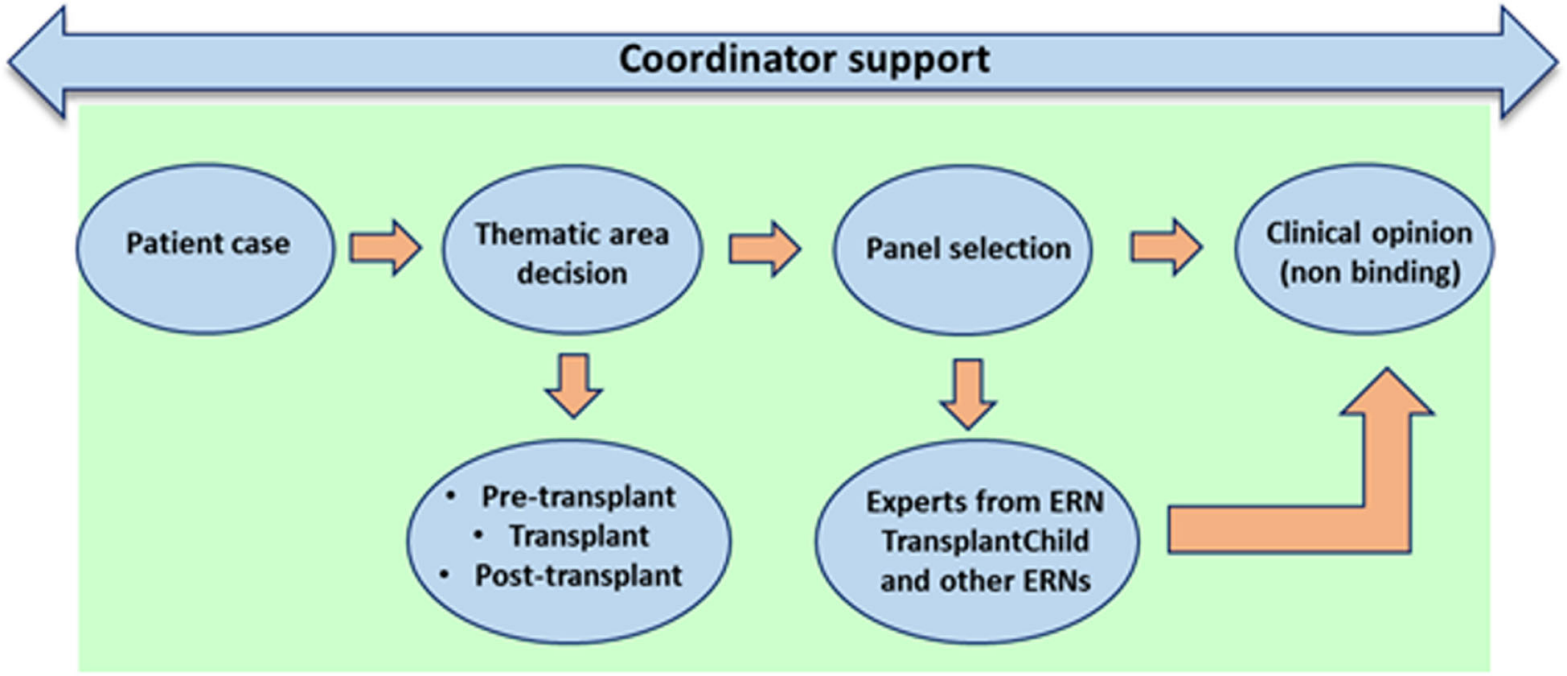
CPMS Virtual Advisory Pathway in the ERN TransplantChild

CPMS works as follow: A challenging case is introduced in the platform. The healthcare provider invites a panel of around 4 to 5 experts from their ERN and also from other ERNs where the expertise will add value to the outcome of the case to make their contribution. Images, genetic information and other data related to the patient, can be uploaded securely. The panel of experts advise on the case in a given period with non-binding clinical opinions (Fig. 3). CPMS collects the following information securely: a) structured patient information (e.g. name, date of birth, gender), clinical data using international standard ontologies; b) upload of digital medical images through Picture Archive and Communication Systems (PACS) in multiple formats for ultrasound (US), magnetic resonance (MR), nuclear medicine imaging, positron emission tomography (PET), computed tomography (CT), endoscopy (ES), mammograms (MG), digital radiography (DR), computed radiography (CR), histopathology; c) anatomical pathology data (macroscopic, microscopic, biochemical, immunologic and molecular examination of organs and tissues); d) genetic and genomic information and pedigree/family history in line with international standards. CPMS encrypts and stores the data in a relational database.

The system also pseudonymises patient data (at both the level of the patient/clinician and at the level of the researcher) after the case is closed and where consent has been provided, stores the data in a low accessibility database and provides a mechanism to allow an ERN to collect and export data for potential sharing, or future use in clinical decision-making tools, protocols, guidelines, case library or research.

### ERN TransplantChild registry

A registry is the best way of pooling data to achieve a sufficient sample size for epidemiological and/or clinical research to ultimately improve health outcomes. Patient registries can form the basis for the development of support networks and national or regional patient advocacy groups where none exists. With this in mind, ERN TransplantChild and supported by the EC, is working in a new registry: paediatric transplantation European registry (PETER). The aim is allow generating real world evidence monitoring by the identification of common outcomes for all types of transplant, and which can be used as a model to support care and research for the benefit of patients, improvement of the transplanted patient healthcare, their life expectancy and long-term QoL of children and their families. PETER will be established following recommendations and standards by the Joint Research Centre in the EU, allowing interoperability with other registries in the EU members’ states, while fully respecting data protection. The integration of clinical knowledge and research information with the patients reported outcomes will constitute an innovative step in clinical decision-making and the patient’s involvement their medical care, and will impact on an improvement in their well-being and health outcomes.

## Conclusions

In comparison with adult transplantation, the lack of robust data related to PT remains problematic, and further research and efforts from a multidisciplinary point of view are needed to develop safer and more effective treatments and to improve the QoL of this patient population. Additionally, it is crucial to determine the clinical, social and economic impact across Europe of PT which is currently largely concealed. Multidisciplinary and international approaches to the management of PT patients will provide clear benefits at clinical, economic and social levels will create a better understanding of the need and costs of PT in Europe enabling better healthcare planning. It is also important to highlight the need to hear the voices of but also work with and educate families and patients’ support groups recognising that PT is a complex and lengthy process requiring a lifetime of care.

## Data Availability

Not applicable.

## Abbreviations

CPMS: Clinical Patient Management System
CR: Computed radiography
CT: Computed tomography
DR: Digital radiography
ERN: European Reference Networks
ES: Endoscopy
EU: European Union
HRQoL: Health-related quality of life
HSCT: Haematopoietic stem cell transplant
MG: Mammograms
MR: Magnetic resonance
PACS: Picture Archive and Communication Systems
PET: Positron emission tomography
PETER: Paediatric transplantation European registry
PT: Paediatric Transplantation
QoL: Quality of Life
SOT: Solid Organ Transplantation
US: Ultrasound
WG: Working Group
